# Hepatoprotective effect of silymarin on fructose induced nonalcoholic fatty liver disease in male albino *wistar* rats

**DOI:** 10.1186/s12906-021-03275-5

**Published:** 2021-03-30

**Authors:** Tewodros Mengesha, N. Gnana Sekaran, Tsegaye Mehare

**Affiliations:** 1 Department of Biomedical Science, College of Medicine and Health Science, Dilla University, Dilla, Ethiopia; 2 Department of Biochemistry, School of Medicine, Addis Ababa University, Addis Ababa, Ethiopia; 3 Department of Biomedical Science, College of Medicine and Health Science, Dilla University, Dilla, Ethiopia

**Keywords:** Nonalcoholic fatty liver disease, Silymarin, Lipid peroxidation, Dyslipidemia, Total antioxidant status, Reduced glutathione

## Abstract

**Background:**

Nonalcoholic fatty liver disease (NAFLD) is one of the most common causes of chronic liver disease in the Western world, and it’s likely to parallel the increasing prevalence of type 2 diabetes, obesity, and other components of metabolic syndrome. However, optimal treatment for NAFLD has not been established yet. Therefore, this study investigated the hepatoprotective effect of silymarin on fructose-induced nonalcoholic fatty liver disease in rats.

**Methods:**

Thirty male Wistar rats were randomly divided into five groups; normal control group that consumed tap water, silymarin control group that consumed tap water and silymarin (400 mg/kg/day), fructose control group that consumed 20% fructose solution, treatment group that consumed 20% fructose solution and silymarin (200 mg/kg/day), and another treatment group that consumed 20% fructose solution and silymarin (400 mg/kg/day). Hepatic triglyceride, serum lipid profile, lipid peroxidation, antioxidant level, morphological features, and histopathological changes were investigated. The data were analyzed using one-way analysis of variance (ANOVA) followed by Tukey multiple comparison test. Statistical significance was determined at p < 0.05.

**Results:**

This study showed that the fructose control group had a significantly high value in the stage of steatosis grade, hepatic triglyceride, serum triglyceride, total cholesterol, low-density lipoprotein cholesterol, alanine aminotransferase, aspartate aminotransferase, and hepatic malondialdehyde concentration as compared to the normal control. However, significantly low values of reduced glutathione and plasma total antioxidant capacity were found. The altered parameters due to fructose drastic effect were ameliorated by silymarin treatment.

**Conclusions:**

The fructose control group developed dyslipidemia, oxidative stress, and mild steatosis that are the characteristics features of NAFLD. However, silymarin-treated groups showed amelioration in oxidative stress, dyslipidemia, and steatosis.

## Background

Non-alcoholic fatty liver disease (NAFLD) is one of the most common causes of chronic liver disease in the Western world, and its prevalence is likely to parallel the increasing prevalence of diabetes, obesity, and other components of metabolic syndrome [1]. NAFLD has a wide spectrum of liver damage which ranges from simple steatosis to inflammation and then to non-alcoholic steatohepatitis (NASH), fibrosis, and cirrhosis [2]. NAFLD is now thought of as the hepatic manifestation of metabolic syndrome and is by now regarded as one of the most common liver diseases worldwide. It is estimated that about 20–30% of the general adult population of most Westernized countries have hepatic steatosis and that 2–3% of adults even suffer from NASH [3, 4].

Fatty liver disease (FLD) is a growing public health problem worldwide. The global prevalence of NAFLD based on a meta-analysis study showed about 25.24% with the highest prevalence in the Middle East and South America and lowest in Africa. Metabolic comorbidities associated with NAFLD included obesity (51.34%), type 2 diabetes (22.51%), hyperlipidemia (69.16%), hypertension (39.34%) and metabolic syndrome (42.54%) [5].

There is growing evidence in both animal models and human studies suggesting that high dietary intake of fructose is an important nutritional factor in the development of the metabolic syndrome and its associated complications. It was shown that fructose overconsumption in humans’ leads to dyslipidemia and ectopic lipid deposition; along with increased hepatic insulin resistance (IR) [6, 7]. Calorically sweetened beverage intake has been related to the risk of NAFLD. The increase in plasma triglyceride concentrations by sugar-sweetened beverages can be attributed to fructose rather than glucose in sugar [8].

Silymarin is a natural compound that is present in species derived from *Silybum marianum*, which is commonly known as milk thistle. Silymarin belongs to the Aster family (Asteraceae or Compositae). The mature plant has large brilliant purple flowers and abundant thorns. The plant grows in places with sufficient sun exposure [9]. Silymarin is a complex mixture of flavonolignan isomers, namely silybin, isosilybin, silydianin and silychristin with an empirical formula C_25_H_22_O_10_. Its active constituents are collectively known as silymarin [10]. The plant contains at least seven flavolignans and the flavonoid taxifolin. The most important flavolignans present include silybin, silydianin, and silychristin. Silybin represents between 50 and 70% of the extract from silymarin. Silymarin has been used worldwide for many years as a complementary alternative medicine because of the beneficial effects associated with the treatment of hepatic diseases [11].

A study was done to assess the effects of metformin, pioglitazone, and silymarin on the treatment of NAFLD showed that all drugs are beneficial in improving biochemical indices in patients with NAFLD. However, changes in aspartate aminotransferase (AST) and alanine aminotransferase (ALT) in silymarin group showed more improvement than the other groups and the average difference between changes were significant between silymarin and metformin group [12]. A study done on an open-label, prospective randomized study to compare the therapeutic effects of silymarin and vitamin E in NAFLD at the end of the 12-week treatment period showed that there was a significant decrease in the serum AST and ALT levels in both treatment groups. The decrease in AST level in the *Silybum marianum* group as compared to the vitamin E group was significant. In general *Silybum marianum* and vitamin E treatment appeared to be significantly effective in the biochemical improvement and decreasing the ALT and AST levels in patients with NAFLD [13, 14].

Furthermore, several groups of drugs have been suggested according to the pathomechanisms of liver injury in NASH; including antioxidants, carnitine, and insulin sensitizers. However, while some agents showed modest improvements in liver function tests (LFTs) and even histologic parameters, the agents mentioned above are generally used to modify risk factors than as primary therapy for NASH [15]. Therefore in this study, we investigated the hepatoprotective effect of silymarin on fructose-induced NAFLD.

## Methods

### The study area and period

The study was conducted at Black Lion Collage of Health and Medical Sciences, School of Medicine, Department of Biochemistry Master of Science laboratory, Department of Pharmaceutics and Social study laboratory, and Department of Microbiology and Immunology laboratory from November 2018 to June 2019.

### Ethics statement

The experiment was performed after the protocol was approved by Addis Ababa University, College of Health and Medical Science, Department of Biochemistry Research and Ethical Review Committee meeting number DRERC: 06/18 with a protocol number of 9/18 following the code of ethics of animal experiments which comply with national and international scientific and ethical guidelines.

### Study animals

Thirty male Wistar Albino rats weighing (151–170 g) were obtained from the animal experiment center of the Ethiopian Public Health Institute. Animals were maintained at 23 ± 1 °C room temperature and 12/12 h dark/light rhythm. The rats were acclimatized for two weeks before the experiment started. Female rats were excluded from the study because of their cyclic hormonal variations.

### Treatment protocol and animal grouping

The experimental rats were randomly divided into five groups consisting of 6 rats each group. The experimental animals in different groups were as follows:

- Normal control group (NC) - a group that took standard chow & water only.
- Fructose control group (FC) **-** a group that took 20% fructose solution and standard chow.
- Silymarin control group (SC) - treatment control group that took standard chow, water and 400 mg/kg silymarin orally as a treatment.
- Fructose + Silymarin (200 mg/Kg) group (FTH) **–** a group that took standard chow, 20% fructose solution and 200 mg/kg silymarin orally as a treatment.
- Fructose + Silymarin (400 mg/Kg) group (FFH**)** – a group that took standard chow, 20% fructose solution and 400 mg/kg silymarin orally as a treatment.

### Drugs/chemicals

Silymarin was purchased from micro labs (Karnataka, India). Most of the reagents were purchased from the Research Lab Fine Chem Industries (Mumbai, India). The kits for total antioxidant capacity, reduced glutathione, and lipid peroxidation parameters were purchased from HiMedia (Mumbai, India). All the chemicals used in the experiments were of analytical grade.

### Preparation of fructose and silymarin

The fructose solution was prepared according to Mamikutty et al [16] by dissolving 20 g pure fructose crystalline purchased from Kibbutz Maanit (Maanit, Israel) in 100 ml of tap water (20% w/v). Silymarin was dissolved in distilled water. The maximum amount of silymarin solution given to the rats was decided by their weight using 20 ml/kg as a reference volume based on the OECD’s (organization of economic corporation and development’s) guidelines [17]. According to the guideline, 10 mg/ml and 20 mg/ml of silymarin were prepared for a lower dose (200 mg/kg) and a higher dose (400 mg/kg) respectively. The dose was established based on the lethal dose (LD-50) according to Radko and Cybulski [18]. Silymarin was administered orally for 8 weeks.

### Biochemical studies

#### Tissue preparation

At the end of the experiment day (after eight weeks), the rats were fasted overnight, anesthetized using light diethyl ether, and the blood was collected through cardiac puncture [19]. After that, the rats were sacrificed by cervical dislocation, and the liver was isolated. The liver was minced with sharp scissors in the proportion of 1:10 (w/v) ice-cold phosphate buffer saline (0.1 M; PH 7.4) [20] and homogenized by using Bio-Gen PRO200 Homogenizer (USA). Then the homogenates were centrifuged for 20 min at 4000 x g at 4 °C. Aliquots of homogenates were used for the determination of hepatic malondialdehyde (H-MDA) and reduced glutathione (H-GSH).

#### Measurement of lipid profiles

Serum lipid profiles were measured with conventional laboratory methods using an auto-analyzer (Mindrey BS-200 Full Chemistry Analyzer, China).

#### Extraction of total lipid and assay of liver triglyceride

To prepare lipid extracts from liver tissues, 0.5 g (wet weight) each of rat liver tissues was homogenized with 10 ml of the chloroform/methyl alcohol mixture (2/1 by volume), and then centrifuged at 2000 rpm for 20 min according to Folch et al [21]. Briefly, the crude extract was mixed thoroughly with 0.2 its volume of normal saline, and the mixture was allowed to separate into two phases, without interfacial fluff by centrifugation at 2400 rpm for 20 min. Then as much of the upper phase as possible was removed by siphoning, and removal of its solutes was completed by rinsing the interface three times with small amounts of pure solvents upper phase in such a way as not to disturb the lower phase. Finally, the lower phase and remaining rinsing fluid were made into one phase by the addition of methanol, and the resulting solution was diluted to any desired final volume by the addition of a 2:1 chloroform-methanol mixture.

After total lipid extraction, simple enzymatic determination of tissue triglyceride was done according to Danno et al [22]. Briefly, evaporate the extraction on a centrifugal concentrator and then redissolved the residue in a small amount of benzene. Transfer this new mixture to a 15 ml falcon tube and diluted to the mark with more benzene. Thereafter, aliquots of the known working standards and the liver lipid extracts in benzene were transferred into test tubes. Then the solvents were evaporated with a centrifugal concentrator and redissolved the triglyceride standards and liver samples in 30 μL of tert-butyl alcohol and 20 μL of the Triton X-100/methyl alcohol mixture. These redissolved materials were mixed carefully. To each test tube, 1.0 ml of enzymatic reagent was added and mixed carefully again. Then the standards, samples, and appropriate blanks were incubated for 18 min at 37 °C and then measured the absorbance at 505 nm vs. a reagent blank on Solar CM 2203 Spectro-fluorometer (Russia).

#### Determination of lipid peroxidation

Lipid peroxide content was estimated according to the method of Ohkawa et al [23]. Briefly, acetic acid detaches the lipid and protein of the tissue. The protein in the reaction mixture was dissolved by the addition of sodium dodecyl sulfate (SDS). 2-thiobarbituric acid (TBA) reacts with lipid peroxide, hydroperoxide, and oxygen labile double bond to form the color products with absorption maxima at 532 nm. In this assay, 0.2 ml of tissue homogenate was mixed with 1.0 ml of 20% acetic acid. Subsequently, 0.2 ml of 8% aqueous SDS was mixed in the above reaction mixture, the pH of the mixture was adjusted at 4.0 using concentrated NaOH solution. After adjusting the pH of the reaction mixture, 1.5 ml of 0.8% TBA solution and a sufficient amount of distilled water were added to a final volume of 4.0 ml. Then the reaction mixture was incubated in a boiling water bath at 37 °C for 1 h. After cooling, 1.0 ml of distilled water and 5.0 ml of butanol/pyridine mixture (15:1 v/v) were added and mixed. The reaction mixture was then centrifuged at 10,000 x g for 15 min. The organic phase obtained after centrifugation was used for measuring the absorbance at 532 nm in Solar CM 2203 Spectro-fluorometer.

#### Measurement of plasma total antioxidant capacity

A direct measurement method for total antioxidant capacity using a new generation, more stable 2,2′-Azino-bis(3-ethylbenzothiazoline-6-sulphonic acid) diammonium salt (ABTS) radical cation was used to determine the total antioxidant capacity (TAC) as previously described by another study [24]. In principle, the reduced ABTS molecule is oxidized to ABTS+ using hydrogen peroxide alone in an acidic medium (the acetate buffer 30 Mm, pH 3.6). In the acetate buffer solution, the concentrate (deep green) ABTS+ molecules stay more stable for a long time. While it is diluted with a more concentrated acetate buffer solution at high pH values (the acetate buffer 0.4 M, pH 5.8), the color is spontaneously and slowly bleached. Antioxidants present in the sample accelerate the bleaching rate to a degree proportional to their concentrations. This reaction can be monitored spectrophotometrically and the bleaching rate is inversely related to the TAC of the sample. The reaction rate is calibrated with Ascorbic acid standard for TAC measurement assays, and the assay results are expressed in mmol ascorbic acid equivalent/L. This procedure was done according to the modified microplate assay (improved method of the total antioxidant assay) for TAC as described before by Gupta et al [25].

#### Estimation of free sulfhydryl group

The free sulfhydryl group was estimated by the method of Ellman [26] as modified by Sedlak et al [27]. Briefly, 5–5′-dithiobis-2-nitrobenzoic acid (DTNB) is reduced by -SH groups of glutathione in alkaline medium to produce one mole of 2-nitro-5-mercaptobenzoic acid per mole of -SH group. Since the anion (2-nitro-5-mercaptobenzoic acid) has an intense yellow color, it can be used to measure the –SH group at 412 nm.

#### Determination of LFTs

At the end of the experimental period, the rats were anesthetized using diethyl ether and blood was drawn from the heart by cardiac puncture and collected into serum separator test tubes (Guangzhou, China) for serum and ethylenediaminetetraacetic acid tubes (EDTA tube) (Guangzhou, China) for plasma. Serum activities of ALT and AST were measured with routine laboratory methods using an auto-analyzer (Mindrey, BS-200 Full chemistry Analyzer, China).

### Histopathological studies

Liver tissues were cut and fixed with 4% paraformaldehyde. The tissue slices were embedded in paraffin. Tissue sections of 5 μm were stained with hematoxylin and eosin [28] and histology results were read by a single independent pathologist, blinded to experimental design and treatment groups using light microscope Leica DM750 (Morrisville, USA). Steatosis, fibrosis, and disease activity score were semi-quantitatively evaluated according to the standard criteria of grading and staging for NAFLD [29].

### Statistical analysis

Data were analyzed using Statistical Package for Social Science (SPSS) software (V-16.00). Data were compared using one-way ANOVA followed by posthoc Tukey’s test to determine significant difference between groups. Frequency data (pathologic grading of the fatty liver) were analyzed. A *p*-values < 0.05 were considered statistically significant.

## Results

### Effect of silymarin on food and liquid intake

**Fig. 1:**
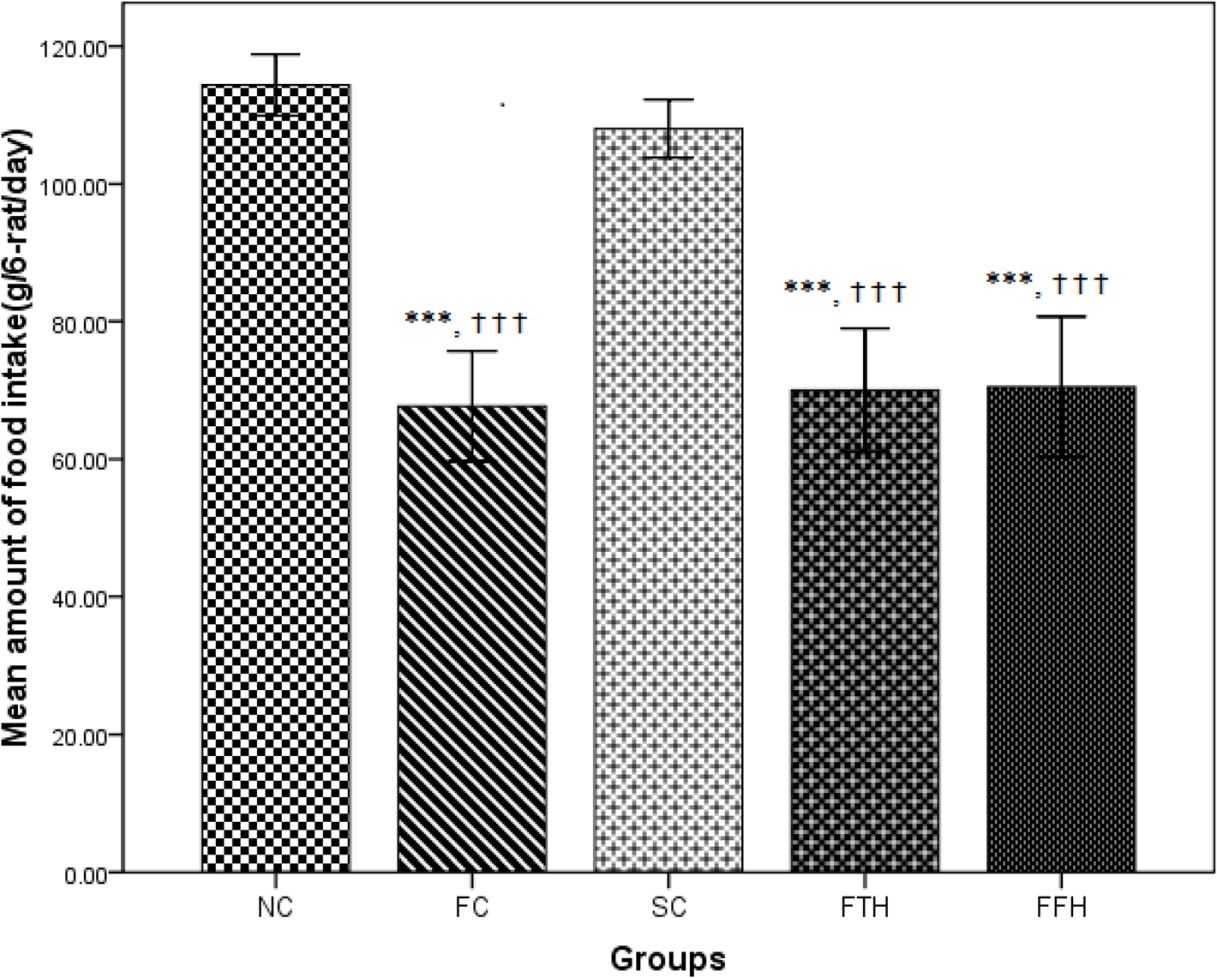
Effect of silymarin on food intake. Food intake were measured and recorded during eight weeks of the experiment time. NC = normal control, FC = fructose control, SC = silymarin control, FTH = fructose + 200 mg/kg silymarin, FFH = fructose + 400 mg/kg silymarin

**Fig. 2:**
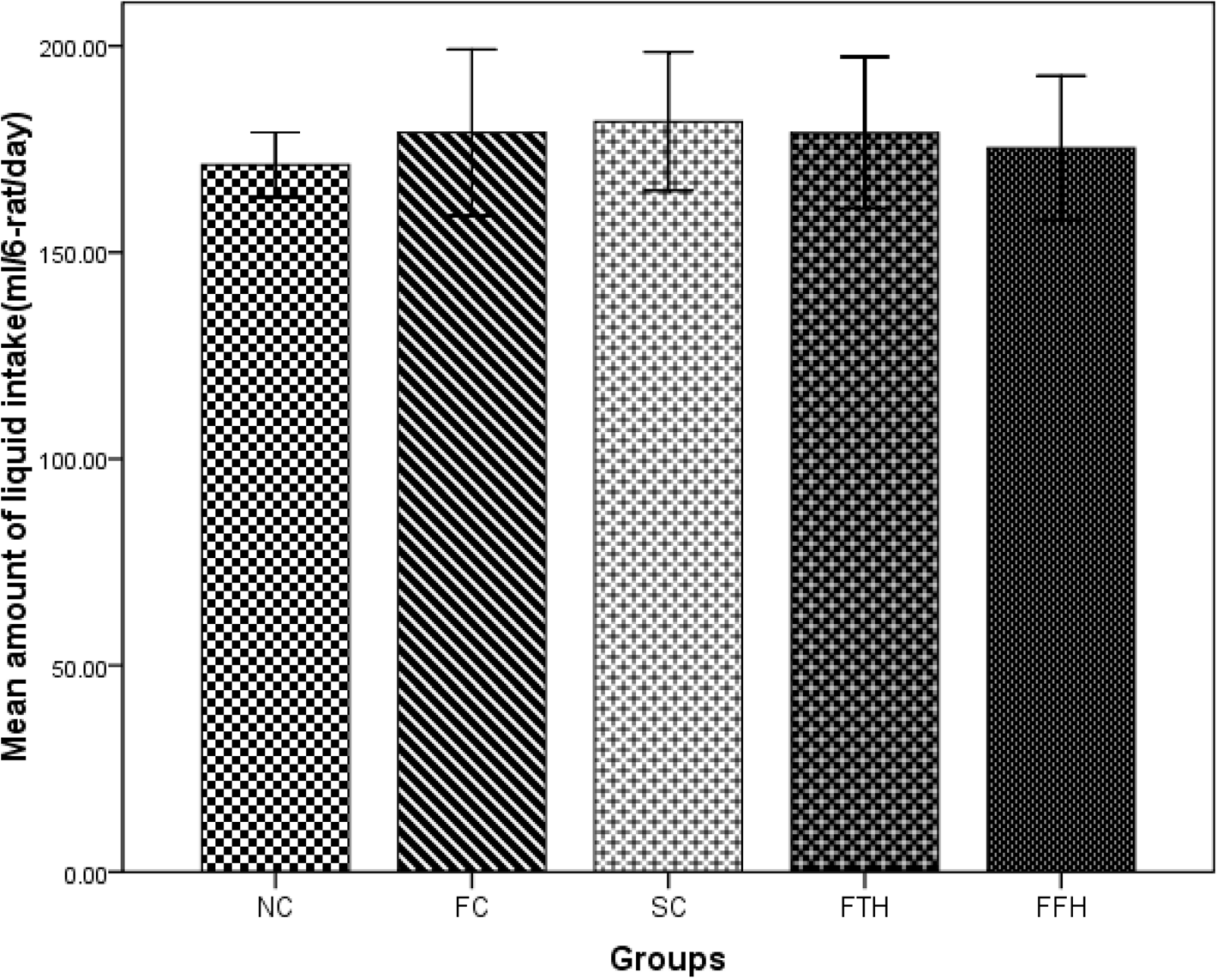
Effect of silymarin on liquid intake. Liquid intake were measured and recorded during eight weeks of the experiment time. NC = normal control, FC = fructose control, SC = silymarin control, FTH = fructose + 200 mg/kg silymarin, FFH = fructose + 400 mg/kg silymarin

The fructose control group consumed less standard chow (67.69 g/day) than the normal control group (114.36 g/day) and the silymarin control group (108.05 g/day) (p < 0.001). Silymarin either 200 mg/kg (70.07 g/day) or 400 mg/kg (70.52 g/day) treated group showed a slightly higher chow consumption as compared to the fructose control group (Fig. 1). The fructose solution and tap water intake did not show any significant difference among groups, unlike their food consumption. The fructose control group had a higher fructose solution intake (179.04 ml//day) than the normal control group (171.27 ml/day) (Fig. 2).

### Effect of silymarin on liver weight, liver weight/body weight, body mass index and body weight gain

The body weight gain and body mass index did not show a significant difference. However, the liver weight was significantly higher (*p* < 0.05) in the fructose control group as compared to the normal control group (Table 1).

**Table 1:** Effect of silymarin on liver weight, liver weight/body weight ratio, body mass index, body weight gain

Group	Weight measurement			
	Liver weight(g)	Liver weight/body weight (g/g)	Body mass index (kg/m^2^)	Body Weight Gain(g)
NC	6.24 ± 0.53	0.025 ± 0.002	5.66 ± 0.18	71.67 ± 27.05
FC	6.98 ± 0.24*	0.029 ± 0.003	5.70 ± 0.39	79.67 ± 19.36
SC	6.34 ± 0.41	0.026 ± 0.002	5.65 ± 0.19	71.83 ± 9.15
FTH	6.71 ± 0.33	0.027 ± 0.003	5.81 ± 0.48	74.33 ± 17.67
FFH	6.50 ± 0.36	0.026 ± 0.004	5.75 ± 0.28	72.67 ± 25.90

At the end of the trial liver weight, liver weight/body weight ratio, body mass index and body weight gain were measured and calculated. *NC* normal control, *FC* fructose control, *SC* silymarin control, *FTH* fructose + 200 mg/kg silymarin, *FFH* fructose + 400 mg/kg silymarin. Data were presented as mean ± SD. *N* = 6 for each group. * *p* < 0.05 vs. NC

### Effect of silymarin on serum and liver lipid profile

The fructose control group showed significantly higher serum total cholesterol (TC) and low-density lipoprotein-cholesterol (LDL-C) as compared to the normal control group (*P* < 0.05). However, their high-density lipoprotein-cholesterol (HDL-C) was lower and not statistically significant. In silymarin-treated groups: TC, LDL-C, and HDL-C levels showed improvement (Table 2). The Hepatic triglyceride (H-TG) and serum triglyceride (S-TG) of the fructose control group presented significantly higher values as compared to the normal control group and the silymarin control group (p < 0.001). Both silymarin-treated groups exhibited significant improvement in H-TG and S-TG (*p* < 0.05) (Table 2).

**Table 2:** Effect of silymarin on serum and liver lipid profile

Group	Lipid Profile of Rats				
	LDL-C (mg/dl)	TC (mg/dl)	HDL-C (mg/dl)	S-TG (mg/dl)	H-TG (mmol/g)
NC	5.50 ± 2.43	39.00 ± 4.77	17.00 ± 2.28	39.17 ± 5.52	0.5 ± 0.01
FC	9.00 ± 2.37*	53.83 ± 7.91*	12.17 ± 3.54	57.83 ± 8.10**	0.57 ± 0.03***
SC	6.00 ± 1.79	42.50 ± 6.95	15.83 ± 3.31	42.83 ± 6.05 ^**##**^	0.52 ± 0.01 ^**###**^
FTH	6.83 ± 1.94	50.67 ± 7.89	12.67 ± 3.08	45.50 ± 9.65 ^**#**^	0.54 ± 0.02 ^**#**^
FFH	6.67 ± 1.21	48.50 ± 10.84	15.33 ± 2.58	44.17 ± 4.96 ^**#**^	0.53 ± 0.02 ^**#**^

At the end of the experiment serum and liver lipid profile were measured. *NC* normal control, *FC* fructose control, *SC* silymarin control, *FTH* fructose + 200 mg/kg silymarin, *FFH* fructose + 400 mg/kg silymarin. Data were presented as mean ± SD. *N* = 6 for each group. * *p* < 0.05 vs. NC; ** *p* < 0.01 vs. NC; *** *p* < 0.001 vs. NC and ^**#**^ *p* < 0.05 vs. FC; ^**##**^ *p* < 0.01 vs. FC; ^**###**^ *p* < 0.001 vs. FC

### Effect of silymarin on hepatic MDA, GSH and plasma TAC

The fructose control group showed a significantly higher hepatic malondialdehyde (H-MDA) as compared to the normal control group (*p* < 0.001). Both silymarin-treated groups were significantly prevented from lipid peroxidation (Table 3). The fructose control group had lower hepatic reduced glutathione (H-GSH) as compared to the normal control group (*p* < 0.01). The 400 mg/kg silymarin-treated group showed a significantly higher H-GSH as compared to the fructose control group (*p* < 0.01). However, the 200 mg/kg silymarin-treated group did not show a statistical difference (Table 3). The fructose control group had low values of plasma total antioxidant capacity (P-TAC) as compared to the normal control group (*p* < 0.01). Both silymarin-treated groups displayed a significantly higher P-TAC (*p* < 0.05) (Table 3).

**Table 3:** Effects of silymarin on H-MDA, H-GSH and Plasma TAC

GROUP	H-MDA (nmol/g tissue)	H-GSH (μmol/g tissue)	P-TAC (nmol AAEAC/L)
NC	52.65 ± 3.49	37.98 ± 1.07	0.933 ± 0.001
FC	67.60 ± 3.21***	33.93 ± 0.91***	0.927 ± 0.002***
SC	56.21 ± 4.99 ^**##**^	36.53 ± 0.89 ^**##**^	0.931 ± 0.002 ^**##**^
FTH	59.77 ± 5.40 ^#^	35.49 ± 0.98	0.930 ± 0.002 ^**#**^
FFH	57.63 ± 4.48 ^**##**^	36.35 ± 0.93 ^##^	0.930 ± 0.001^#^

At the end of the experiment the H-MDA, H-GSH and Plasma TAC were measured. *NC* normal control, *FC* fructose control, *SC* silymarin control, *FTH* fructose + 200 mg/kg silymarin, *FFH* fructose + 400 mg/kg silymarin. Data were presented as mean ± SD. *N* = 6 for each group. *** *p* < 0.001 vs. NC and ^**#**^ *p* < 0.05 vs. FC; ^**##**^ *p* < 0.01 vs. FC

### Effect of silymarin on LFTs

**Fig. 3:**
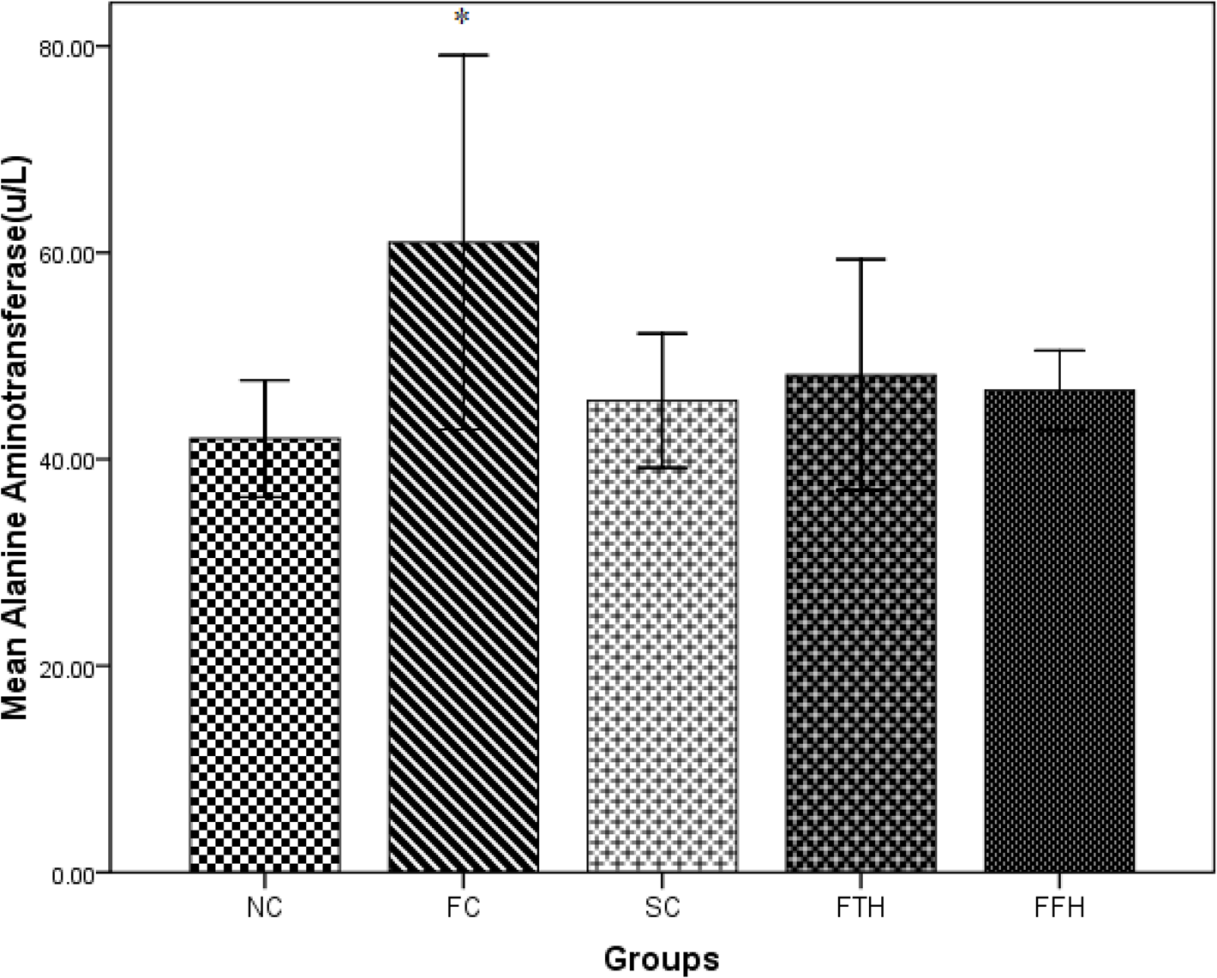
Effect of silymarin administration on serum alanine aminotransferase. NC = normal control, FC = fructose control, SC = silymarin control, FTH = fructose + 200 mg/kg silymarin, FFH = fructose + 400 mg/kg silymarin

**Fig. 4:**
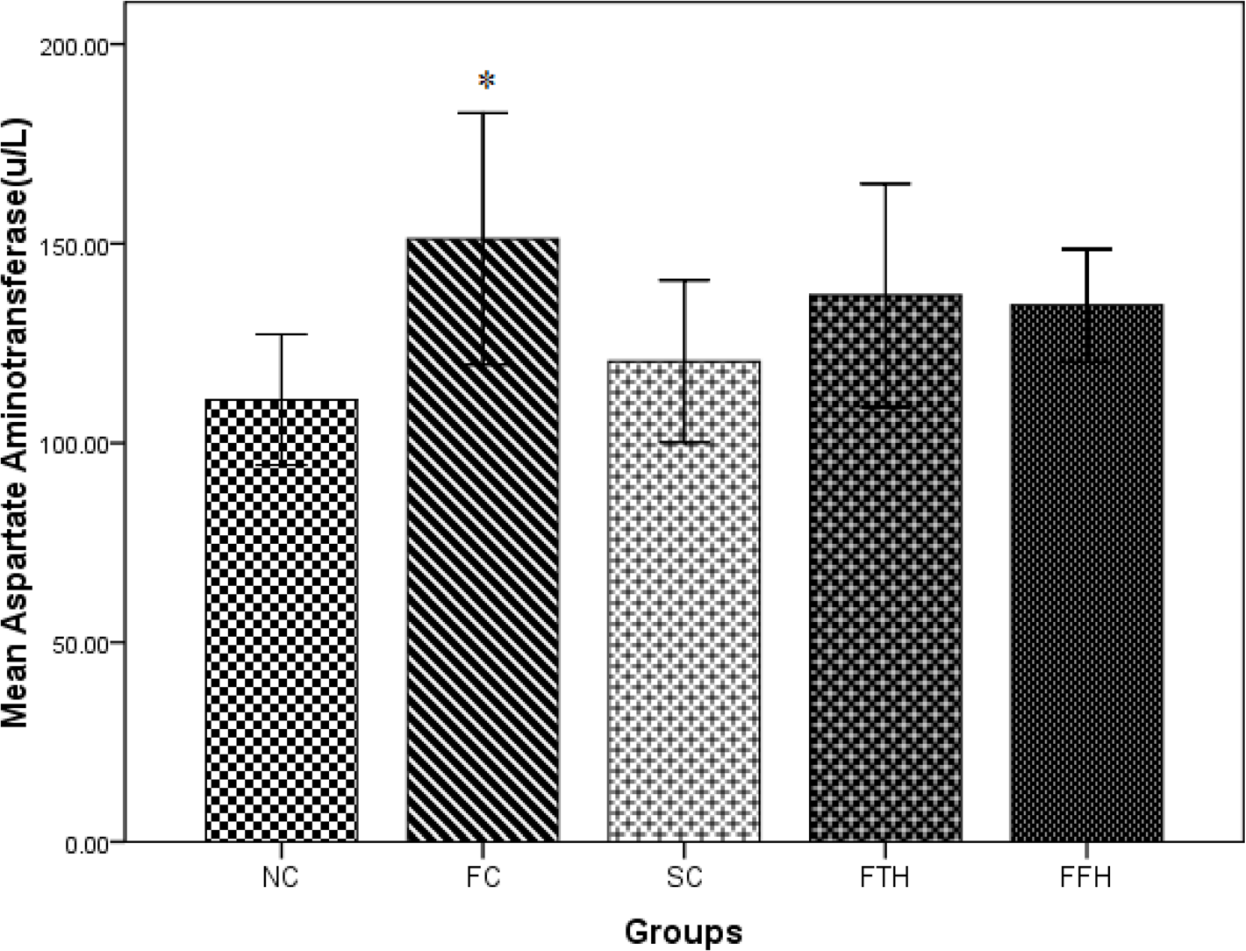
Effect of silymarin on serum aspartate aminotransferase. NC = normal control, FC = fructose control, SC = silymarin control, FTH = fructose + 200 mg/kg silymarin, FFH = fructose + 400 mg/kg silymarin

The fructose control group displayed significantly higher values of serum ALT and AST as compared to the normal control group (*p* < 0.05). Both silymarin-treated groups demonstrated lower values of ALT and AST as compared to the fructose control group (Figs. 3 and 4).

### Effects of silymarin on gross hepatic manifestations

**Fig. 5:**
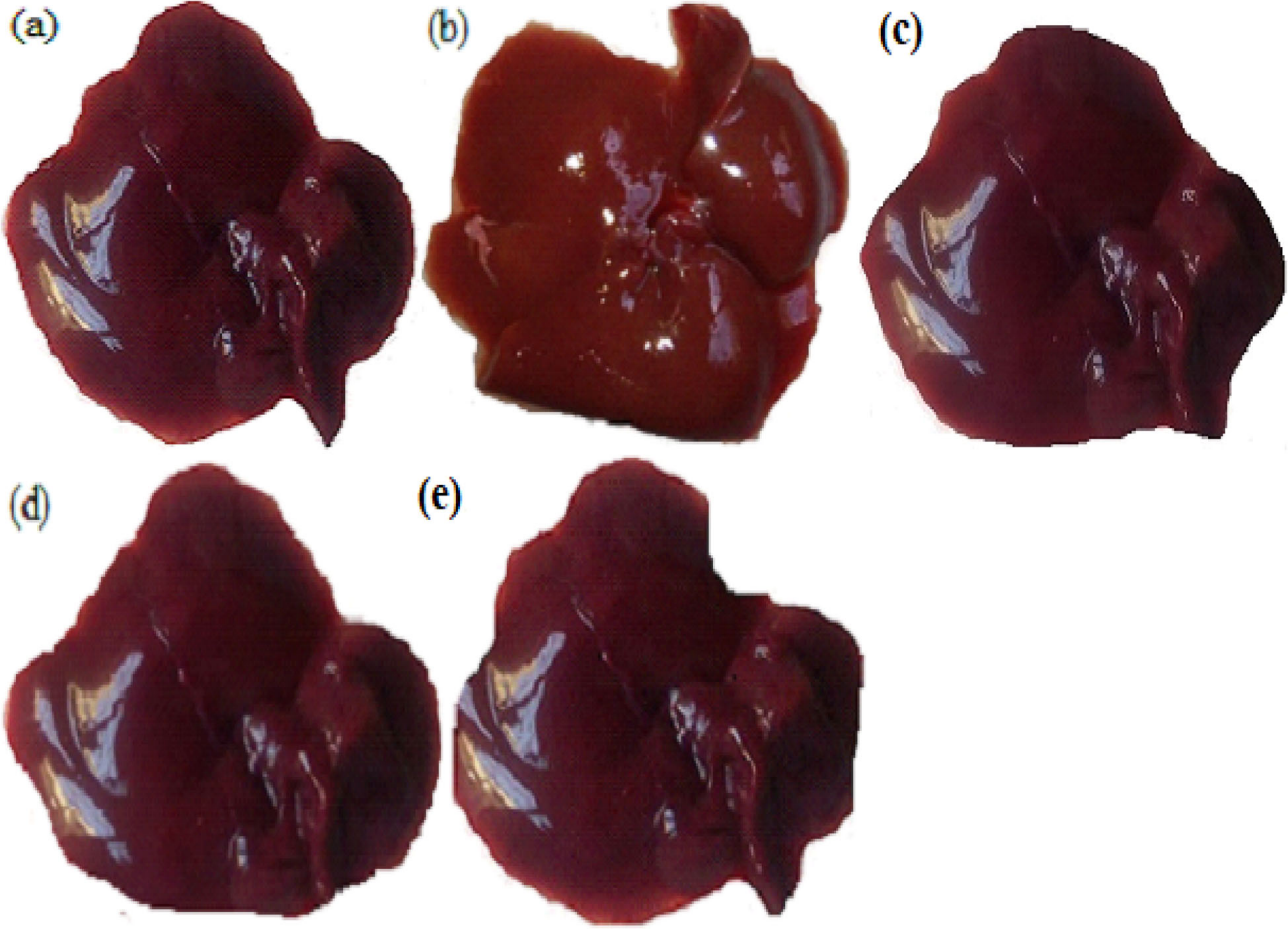
Rat gross hepatic characteristics. NC = normal control, FC = fructose control, SC = silymarin control, FTH = fructose + 200 mg/kg silymarin, FFH = fructose + 400 mg/kg silymarin. **a** NC- with smooth texture and red brown color; **b** FC- rough texture and bright red brown color; **c** SC- red brown color; **d** FTH- red brown color; **e** FFH- red brown color

The liver of the normal control group was of moderate texture, with a smooth surface and red-brown color (Fig. 5a). In contrast, the fructose control group showed an enlarged, bright red-brown color, and relatively hard texture (Fig. 5b). Liver conditions of both silymarin-treated groups demonstrated an intermediate phenotype between those of the above two groups with doomed red-brown color and relatively smooth surface (Fig. 5d and e).

### Effect of silymarin on histopathological manifestations

**Fig. 6:**
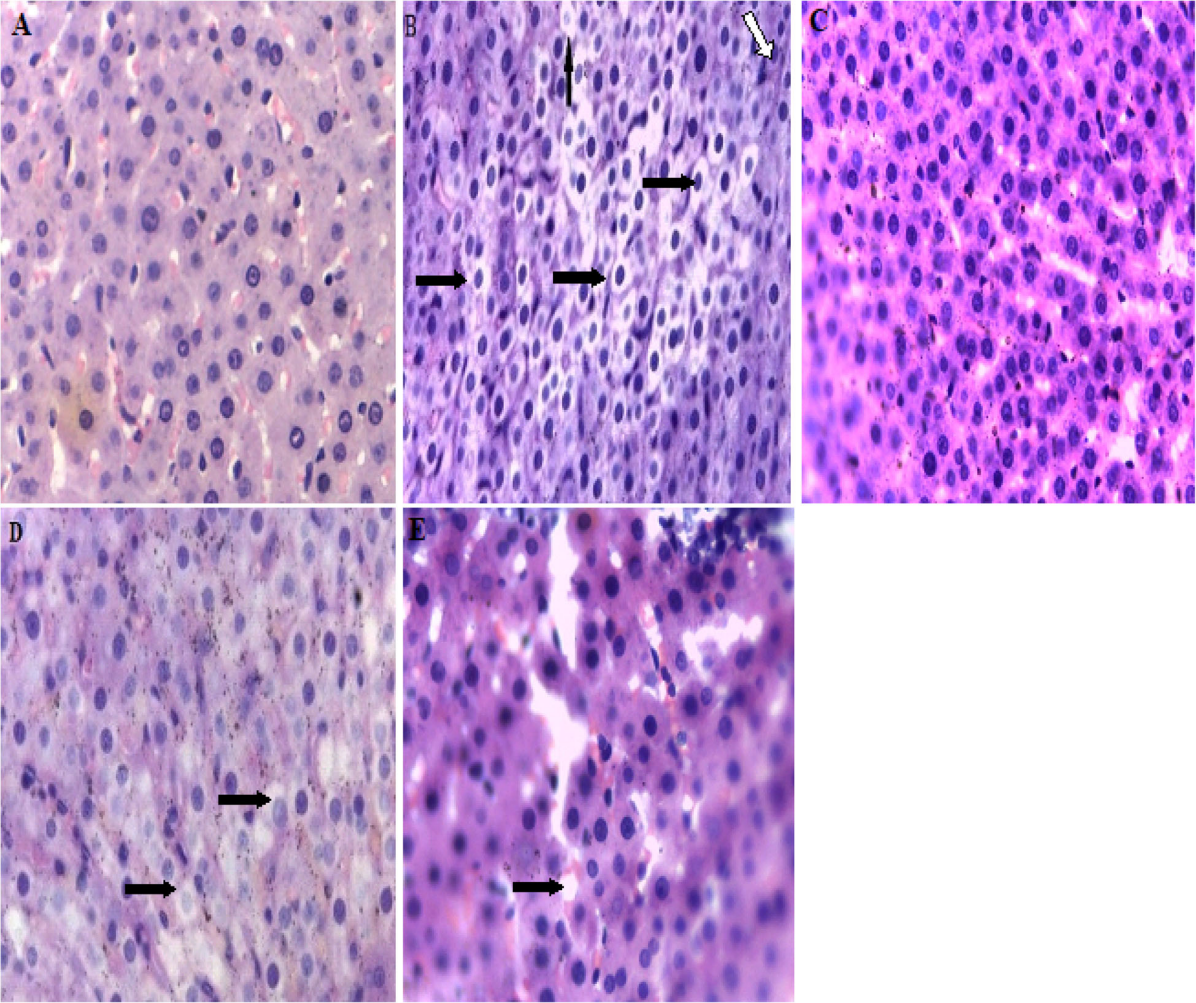
Photomicrographs of liver samples stained with Hematoxylin & Eosin (X40 magnification,  Indicate steatosis,  indicate lobular inflammation). NC = normal control, FC = fructose control, SC = silymarin control, FTH = fructose + 200 mg/kg silymarin, FFH = fructose + 400 mg/kg silymarin. **a** No fatty change in NC group; **b** the micrographs showed steatosis in the FC group; **c** no fatty change on SC group; **d** little improvement of stetosis in FTH group; **e** better improvement of stetosis and lobular inflammation in FFH group. The scale bar is 10 μm in **a, b, c, d** and **e** micrographs

The liver lobules of the normal control group were distinct, and the liver cell cords were arranged regularly (Fig. 6a). However, the fructose control group showed typical steatosis accompanied by a few infiltrating cells (Fig. 6b). The degree of hepatic injury including steatosis, cytological ballooning was attenuated by silymarin (Fig. 6d and e).

### Effect of silymarin on NAFLD scores (NAS)

Based on the NAS showed in (Table 4) below, the fructose control group fulfilled the steatosis score of 1.84. The predominant distribution pattern of the steatosis observed in the fructose control group was in zone 3 (3 rats) as well as in zone 1(3 rats). The silymarin-treated groups were prevented from liver steatosis according to NAS (Table 4).

**Table 4:** Histopathological NAS

NAFLD Activity Score	GROUPS				
	NC	FC	SC	FTH	FFH
Steatosis grade	0	0.83*	0.17	0.50	0.33
Location of steatosis)	0	0.50	0	0	0
Microvesicular steatosis	0	0.17	0	0	0
Lobular inflammation	0	0.17	0	0	0
Fibrosis stage	0	0	0	0	0
Microgranulomas	0	0	0	0	0
Large lipogranulomas	0	0	0	0	0
Portal inflammation	0	0	0	0	0
Liver cell injury (ballooning)	0	0.17	0	0	0
Acidophil bodies	0	0	0	0	0
Pigmented microphages	0	0	0	0	0
Mega mitochondria	0	0	0	0	0
Mallory’s hyaline	0	0	0	0	0
Glycogenated nuclei	0	0	0	0	0
Total sum	0	1.84	0.17	0.50	0.33
Diagnostic classification for NASH	Not steatosis	Possible/border line	Not steatosis	Not steatosis	Not steatosis

Histopathological NAS. *NC* normal control, *FC* fructose control, *SC* silymarin control, *FTH* fructose + 200 mg/kg silymarin, *FFH* fructose + 400 mg/kg silymarin. Values are mean (*n* = 6) for each group. * *p* < 0.05 vs. NC

## Discussion

Our study aimed to evaluate the hepatoprotective effect of silymarin in fructose-induced NAFLD rats. Many studies investigating the influence of diet on liver fat using a high-calorie diet that leads to a significant increase in liver fat content were reviewed elsewhere before [30, 31]. NAFLD is a disease that can be defined by evidence of hepatic steatosis either by imaging or by histology first and secondly confirming whether there are no causes for secondary hepatic fat accumulation such as significant alcohol consumption, use of steatogenic medication, or hereditary disorders [32]. Our study used high fructose consumption as a method to induce NAFLD and silymarin as a protective agent. Generally, the hepatoprotective effect of silymarin could be summarized; as an anti-inflammatory, antioxidant, antiproliferative, antilipidemic, antifibrotic, nuclear expression regulation, mitochondrial membrane stabilization, improved insulin resistance, preserving hepatic mitochondrial bioenergetics, and decreased elevation of AST and ALT in serum as described before [33–38].

In this study, the liquid intake was not statistically significant, and this showed similar findings revealed by Abdulla et al [39]. Our study showed that fructose consumption reduces food intake. This finding was supported by a previously described finding in which fructose in either 20% or 10% showed a significant reduction in food intake [40]. However, another study showed fructose consumption in either 5% or 10% did not reduce food consumption [41]. In our study, silymarin treatment did not bring any significant change in standard chow consumption. This result was also reinforced by another study [42]. Decreased standard chow consumption due to fructose solution might be overall due to fructose’s ability to compensate the daily calorie requirement of the rats.

Our findings showed that fructose consumption for eight weeks did not exhibit a significant change in body weight gain. This finding was in agreement with a study done on the C57BL/6 mouse model that showed the least impact of fructose on body weight gain [43]. But some other studies revealed that fructose had a highly positive impact on body weight gain [44]. Another study also showed that mice fed with HFD for 14 weeks developed significantly higher body weight and silymarin given orally caused the loss of body weight in diet-induced obesity mice to some extent [45]. However, another study also showed that silymarin did not affect body weight and food intake [46].

In our study, the Liver weight of the fructose control group increased significantly as compared to normal control. This finding was supported by another finding which revealed either fructose alone or in combination with a high-fat diet brought a significant increase in liver weight [47]. This might be due to ectopic lipid accumulation in the liver. One study showed that 12 weeks of fat-induced liver weight gain was significantly reduced by 5 weeks of silymarin treatment [48]. However, in our study, silymarin treatment did not show significant improvement in liver weight gain.

In our study, serum TG, LDL-C, and TC were significantly higher and this finding was similar to other studies done on either fructose or fructose with a high-fat diet that showed a significant change in plasma TG, LDL-C, and TC [49, 50]. In our finding, silymarin in either dose did not show significant improvement except on the S-TG level. This finding was supported by a study that showed oral administration of silymarin did not affect the TC concentration of high fructose high cholesterol diet-induced NAFLD. However, it significantly reduced the LDL-C level [51]. Another study revealed that serum TC, TG, and TC values showed improvement in silybin-treated groups [52]. In our study, silymarin treated groups showed significantly low H-TG level. Another study also reinforced our finding [53].

The fructose control group showed significantly higher ALT and AST levels in this study and these were considered biomarkers for liver injury. Other previous findings were in agreement with this result [54, 55]. In our study, both doses of silymarin treatment brought improvement in serum ALT and AST concentrations. Other studies also showed similar results in different toxic and high fat diet-induced liver injury when treated with silymarin [56].

In our study, elevated levels of H-MDA, reduced levels of H-GSH, and TAC of the plasma were observed in the fructose control group. This finding was also indicated in a previous study [57]. Another study on fructose-sweetened liquid showed the use of products of lipid peroxidation as markers of oxidative stress [58]. Fructose produces damaging effects in hepatocytes because it is highly reactive as a reducing agent and a precursor of advanced glycation end product (AGE). The liver promotes the removal of high levels of fructose aggressively from the bloodstream to prevent the damaging effects of glycation/fructation on serum lipids and proteins. Glycated/fructated proteins not only show impaired functions but are also more susceptible to oxidative damage. Thus proteins are ultimately converted into toxic AGEs [59]. In our study, silymarin-treated groups significantly reduced H-MDA while increased H-GSH and TAC of plasma. This finding was supported by previous studies [60–62].

The normal control group had a relatively small size, smooth texture, and red-brown liver. The fructose control group had a large, hard texture, and bright red-brown liver. The relative reduction in brightness and size was observed in both silymarin-treated groups. This finding was similar to a previous study [63]. The size increment in the liver might be due to the higher numbers of lipid droplets deposited in the hepatocyte cytoplasm.

The histopathological finding showed the ectopic lipid accumulation in the liver and this might be due to fructose overconsumption which led to de novo lipogenesis. Both doses of silymarin prevented liver steatosis in our finding. This finding was in agreement with a study done on hepatoprotective effect of silymarin on different diet combinations to induce steatosis [64].

## Conclusions

This study concluded that high fructose consumption caused the development of dyslipidemia, oxidative stress, and steatosis which are the characteristics feature of NAFLD. These problems were ameliorated through silymarins treatment by improving the liver function and lipid profile panels. Fructose-induced NAFLD was prevented by silymarin via inhibition of lipid peroxidation through a regulatory property of membrane integrity and their oxidant scavenging activity through increased intracellular glutathione level. Silymarin treatment with a higher dose (400 mg/kg) had better efficacy than a lower dose (200 mg/kg) on treating NAFLD.

## Data Availability

The datasets used and/or analyzed during the current study are available with the corresponding author and accessible per reasonable request.

## Abbreviations

ABTS: 2,2′-Azino-bis (3-ethylbenzothiazoline-6-sulphonicacid) diammonium salt
AGE: Advanced Glycation End Product
ALT: Alanine Aminotransferase
ANOVA: Analysis of Variance
AST: Aspartate Aminotransferase
DNL: De Novo Hepatic Lipogenesis
DTNB: 5–5′-dithiobis-2-nitrobenzoic acid
DRERC: Department of Biochemistry Research and Ethical Review Committee
EDTA: Ethylenediaminetetraacetic acid
FLD: Fatty liver disease
GSH: Reduced Glutathione
HDL-C: High Density Lipoprotein Cholesterol
HE: Hematoxylin and Eosin
HFCS: High Fructose Corn Syrup
HFD: High Fat Diets
HCHF: High Carbohydrate High Fat
IR: Insulin Resistance
LD: Liver Disease
LDL: Low Density Lipoprotein
MetS: LFTs Liver function tests Metabolic Syndrome
MDA: Malondialdehyde
NAFLD: Nonalcoholic Fatty Liver Disease
NAS: Nonalcoholic fatty liver disease Activity Score
NASH: Non-Alcoholic Steatohepatitis
OECD: Organization of economic corporation and development’s guidelines
SDS: Sodium dodecyl sulphate
SPSS: Statistical Package for Social Science
TAC: Total Antioxidant Capacity
TAG: Triacylglycerol
TC: Total cholesterol
TCA: TriChloro Acetic Acid
TBA: 2-thiobarbituric acid
TBARS: Thiobarbituric Acid Reactive Substance
VLDL: Very Low Density Lipoprotein
