# Correction to: Hepatoprotective effect of silymarin on fructose induced nonalcoholic fatty liver disease in male albino *wistar* rats

**DOI:** 10.1186/s12906-021-03313-2

**Published:** 2021-05-12

**Authors:** Tewodros Mengesha, Natesan Gnanasekaran, Tsegaye Mehare

**Affiliations:** 1 Department of Biomedical Science, College of Medicine and Health Science, Dilla University, Dilla, Ethiopia; 2 Department of Medical Biochemistry, School of Medicine, College of Health Sciences, Addis Ababa University, Addis Ababa, Ethiopia

**Correction to: BMC Complement Med Ther 21, 104 (2021)**

**https://doi.org/10.1186/s12906-021-03275-5**

Following publication of the original article [1], the authors reported an error in author name, affiliation, and in the Authors’ contributions statement. Authors’ contributions statement should read as below:

**Authors’ contributions**

This work was carried out in collaboration between all authors. TMR designed the study, performed the experiments, searched the literature, collected the data and wrote the manuscript. TM, review the data and give feedback on the article done. NG wrote the research protocol, provided the chemicals for the experiments and supervising the research work, structured the thesis and review the article. All authors read and approved the final manuscript.

The original article [1] has been updated.
